# Experiences and needs of Dutch cancer survivors regarding lifestyle counselling: a qualitative study

**DOI:** 10.1186/s12885-025-15186-6

**Published:** 2025-11-12

**Authors:** Bente van Aken, Anna Manshanden, Willemieke Kroeze, Nadine Florisson, Barbara Groot-Sluijsmans, Meeke Hoedjes, Kristel van Asselt, Lies ter Beek, Ingrid Steenhuis

**Affiliations:** 1 Department of Nursing, Christian University of Applied Sciences Ede (CHE), PO box 80, Ede, 6710 BB The Netherlands; 2 Health and Society group, Department of Social Sciences, Wageningen University & Research, Wageningen, the Netherlands; 3 Department of Health Sciences, Faculty of Sciences, Vrije Universiteit Amsterdam, Amsterdam, The Netherlands; 4 Amsterdam Public Health Research Institute, Amsterdam, The Netherlands; 5 Department of Health, Sport and Physical Activity, Applied University of Amsterdam, Amsterdam, The Netherlands; 6 CoRPS—Center of Research on Psychological and Somatic Disorders, Department of Medical and Clinical Psychology, Tilburg University, Tilburg, The Netherlands; 7 Department of General Practice and Nursing science, Julius Center for Health Sciences and Primary Care, University Medical Center Utrecht, Utrecht University, Utrecht, The Netherlands

**Keywords:** Cancer survivors, Lifestyle counselling, Integrated lifestyle intervention, Qualitative study, Health literacy

## Abstract

**Background:**

Cancer survivors face various short- and long-term consequences of their disease and treatment, which may negatively impact their quality of life. Healthy lifestyle changes can have a positive effect on these consequences but current counselling does not sufficiently meet their needs. This study explores the experiences and needs of cancer survivors regarding lifestyle counselling.

**Methods:**

A qualitative design comprising semi-structured interviews was used. We conducted 18 interviews with Dutch adult cancer survivors with various types of cancer, including people with limited health literacy. The data were analysed using reflexive thematic analysis. This study is part of the GLINK project, which aims to develop and evaluate an integrated lifestyle intervention for cancer survivors.

**Results:**

Dutch cancer survivors experienced current lifestyle counselling as fragmented and not structurally embedded, which requires them to be proactive in seeking support. Participants expressed a desire for stronger integration of lifestyle counselling within oncological (after)care, with clear information on available options and improved accessibility in terms of location, contact with professionals, and referral pathways. They reported a need for personalised, flexible support from professionals specialized in oncology, focusing on individual needs to ensure that patients feel seen and heard.

**Conclusions:**

This study provides insights into how lifestyle counselling for cancer survivors can be improved from the patients’ perspective. These findings can enhance the initiation of lifestyle conversations and referral processes and serve as a foundation for developing an integrated lifestyle intervention for cancer survivors.

**Supplementary Information:**

The online version contains supplementary material available at 10.1186/s12885-025-15186-6.

## Background

The worldwide incidence of cancer cases is estimated to increase from 20.0 million in 2022 to 32.6 million in 2045 [1]. An increasing number of individuals are living with the short- and long-term consequences of cancer, primarily due to population growth and aging [2]. This group is widely defined as cancer survivors, people who have been diagnosed with cancer, including before, during and after treatment [3]. Similarly, in the Netherlands, the number of cancer survivors increased from 800,000 in 2018 to 925,000 in 2023 [4]. Cancer survivors may face physical, psychological, social, and financial consequences, such as fatigue or stress [5–7]. These consequences significantly affect their quality of life [8], defined as a subjective, multi-dimensional concept that reflects how individuals perceive their physical, emotional, social and spiritual well-being, and functional status [9]. In addition, these consequences also place burdens on cancer survivors’ families, the broader community, and healthcare systems [10, 11].

Adopting a healthy lifestyle can help manage the short- and long-term consequences of cancer and its treatment. A number of systematic reviews and meta-analyses have shown the potential of a healthy lifestyle, including diet, physical activity, smoking, weight management, mindfulness, and psychotherapy, to improve quality of life in cancer survivors across various types of cancer [12–15]. In addition to quality of life improvements, healthy lifestyle changes have also been shown to reduce cancer morbidity, mortality and recurrence rates among cancer survivors [16, 17]. Additionally, healthy lifestyle changes positively influence the cardiac risk profile, which is a valuable added benefit given the elevated risk of cardiovascular disease in cancer survivors [18]. Taken together, these findings underscore that adopting and maintaining a healthy lifestyle is critical for cancer survivors, as it can mitigate short- and long-term consequences of treatment, lower recurrence and comorbidity risks, and enhance health outcomes and quality of life.

Although the American Cancer Society and the World Cancer Research Fund provide lifestyle guidelines for cancer survivors, focusing on nutrition, physical activity and weight management [3, 17], systematic reviews reveal that adherence to lifestyle recommendations among cancer survivors is low [19, 20]. Studies have shown that lifestyle change among cancer survivors is influenced by a variety of individual, interpersonal and environmental factors. Barriers to lifestyle change amongst cancer survivors include limited awareness of the link between lifestyle and cancer, perceived lack of evidence or clarity of evidence of lifestyle guidelines, perceptions of lifestyle change as unnecessary and physical challenges such as fatigue, pain and weight-related limitations. In addition, time constraints, cultural beliefs, financial constraints, unmet information needs, limited access to personalised counselling, a lack of motivation and low self-efficacy hinder lifestyle change among cancer survivors [21–24]. Facilitators include advice from and discussion with healthcare professionals, support from relatives and cancer survivor peers, structured education programs, and the use of healthy lifestyle changes as coping strategies to manage side effects and improve well-being [21–24].

The role of healthcare professionals in promoting a healthy lifestyle is important for lifestyle change among cancer survivors [25]. Lifestyle counselling refers to a supportive approach in which healthcare professionals or coaches support individuals to adopt and maintain healthy lifestyle behaviours. It is yet to be decided where lifestyle counselling could best be offered: in oncology follow-up clinics, primary care, or specialized services. In line with this finding, a recent survey study by the Dutch federation of Cancer Patient Organizations found that while most cancer survivors expressed a desire to discuss lifestyle with a healthcare professional, these conversations often did not occur [26]. This gap suggests that despite clear interest among Dutch cancer survivors, the provision of lifestyle advice and referral to lifestyle counselling by healthcare professionals remains insufficient. One possible explanation, as identified by a qualitative study in Australian cancer care [27], is that healthcare professionals face barriers including limited knowledge and confidence, lack of funding for counselling, and complex referral processes.

Cancer survivors with limited health literacy, defined as ‘the degree to which individuals have the capacity to obtain, process, and understand basic health information and services needed to make appropriate health decisions’ [28] are at an additional disadvantage in changing their lifestyle. This is because the increasing complexity of cancer care requires skills and greater involvement from patients and their support system to manage treatments and consequences of cancer [29]. Limited health literacy is related to poorer post-treatment physical activity [30]. Furthermore, several studies show that cancer survivors with limited health literacy experience a reduced quality of life compared to cancer survivors with higher health literacy [31]. Health literacy variety in cancer populations is indicated to be similar to general populations [29], with over one-third of adults in the Netherlands having inadequate or limited health literacy and more common among individuals with practical education levels [32].

Despite the benefits of a healthy lifestyle and the existence of lifestyle guidelines, many cancer survivors and healthcare professionals may not be fully aware of these recommendations, and cancer survivors often face many barriers in lifestyle adaption. This shows there remains a gap in the translation to healthy lifestyle changes of cancer survivors. In the Netherlands, counselling often focuses solely on nutrition and physical activity [26], and is mainly provided monodisciplinary [33]. Improving counselling that aligns the needs of cancer survivors, requires better understanding and addressing the specific lifestyle counselling requirements of cancer survivors, including those with limited health literacy. The aim of this study is to explore the experiences and needs of cancer survivors regarding lifestyle counselling, irrespective of their type of cancer. By gaining deeper insights into their perspectives, this research will aid the development of a tailored integrated lifestyle intervention that meets the needs of people living with the short- and long-term consequences of cancer and its treatment.

## Methods

### Design

This study is part of the Dutch research project ‘GLINK’ (Dutch acronym for Combined Lifestyle Intervention After Cancer), which aims to develop and evaluate an integrated lifestyle intervention for cancer survivors, in co-creation with patients and healthcare professionals. In this study, we explored experiences and needs of patients by using a qualitative study design comprising semi-structured individual interviews. We used an interpretative approach as we aimed to understand patients’ perspectives and experiences [34].

### Participant inclusion

Cancer survivors were recruited based on the following inclusion criteria: adults (age above 18), speaking Dutch, living in the Netherlands, under treatment or treated for cancer, with no maximum of years since their treatment. We chose to include participants with various types of cancer, as they all may experience barriers for lifestyle change and could benefit from positive changes [21–23]. Recruitment continued until a varied group of participants was included. Cancer patients in the terminal phase were excluded from the study. We aimed to ensure variation in gender (male/female), age, educational level, geographical location, type of cancer, type of treatment, and level of health literacy. Dutch educational levels were classified as Vocational Education and Training (VET; Dutch: MBO), University of Applied Sciences (UAS; Dutch: HBO), and Research University (RU; Dutch: WO). In line with our reflexive thematic analysis approach, we did not aim for data saturation. Instead, we guided our sample size using the principles of conceptual coherence and information power, focusing on the richness and diversity of data [35]. Based on this approach, we conducted 18 interviews, which we considered sufficient to capture a rich and diverse range of perspectives.

Our recruitment strategies included purposeful recruitment, network sampling, and snowball sampling [34]. Participants were invited for an interview through distribution of research information flyers via multiple channels, including social media platforms of the researchers, research consortium partners and healthcare professionals in their network, community centres, local cancer support centres, cancer care institutes and patient federation organizations. In addition, interested patients were encouraged to share the flyer within their personal networks. These flyers were written at B1 intermediate language level [36] to ensure accessibility.

After 12 interviews, additional efforts were made to engage more individuals with limited health literacy. Healthcare professionals were informed of this focus and the flyer was adapted to explicitly invite people with practical education, which is associated with limited health literacy [32]. A researcher-identified subjective estimation of health literacy level was made using an adapted checklist from Pharos, the Dutch Center of Expertise on Health Disparities (Additional file 1).

Participants could contact the researcher (BvA) via e-mail or phone to ask questions and to show interest in participation. Subsequently, most participants received the information letter and informed consent form by email, both written at B1 language level and reviewed by a language ambassador with experience in low literacy. In some cases, when preferred by participants, the information letter and informed consent form were introduced and explained at the start of the interview. All interviews were scheduled by telephone. Patients were offered a voucher of 20 euros for participating in the study and were reimbursed for travel expenses if necessary.

### Context: Dutch healthcare for cancer survivors

In the Netherlands, survivorship care is mainly hospital-led. The duration of hospital care and follow-up varies and depends on the cancer site. Referrals for mental support or rehabilitation can be arranged by healthcare professionals from the hospital, like medical specialists or (specialized) nurses, or by primary care professionals such as the general practitioner or oncology nurses. Referrals for rehabilitation may include (oncology specialized) physiotherapists, dietitians, or psychologists. While some support is covered by mandatory basic health insurance, other support, for example physiotherapy, often require supplementary insurance. This may pose financial barriers for some individuals. In some regions, cancer support centres offer accessible, low-threshold support for cancer survivors and their families, including peer counselling, activities, and informational sessions [37].

### Data collection

The interviews were conducted between June and September 2024. To minimize the burden on participants, interviews were scheduled to last no longer than one hour and took place at a location preferred by the participant, either online via Microsoft Teams (*n* = 10) or in-person (*n* = 8). The in-person interviews were conducted at institutions affiliated with the researchers (*n* = 4) or at the participant’s home (*n* = 4). In two interviews, participants’ partners were present and contributed minimal information, which was in line with participants’ answers, and thus included in the coding. Prior to each interview, BvA or NF made initial contact with each participant via telephone to introduce the study and to gather information regarding their type of cancer, cancer- and treatment-related symptoms, and effects of cancer and its treatment on daily lives of participants. Personal characteristics such as age, level of education and place of residence were also asked for during this conversation. All interviews were conducted by BvA and lasted on average 60 min.

A semi-structured interview guide (Additional file 2) was developed for the interviews, inspired by the needs assessment of the Intervention Mapping (IM) approach, the Health Belief Model (HBM) and the concept of Positive Health, while also drawing on expert opinion and experiences of the research team. IM [38] is a systematic approach for development of health promotion programs. The first step of IM involves a needs assessment to assess experiences, values, needs and preferences of cancer survivors regarding lifestyle counselling. In this needs assessment, we utilized constructs from the HBM [39] that predict a healthy lifestyle, including perceived susceptibility of disease recurrence, severity of the disease including physical and social consequences, benefits and barriers of adopting a healthy lifestyle, cues to action and self-efficacy. Additionally, Positive health, as defined by Huber et al. [40] refers to ‘the ability to adapt and to self-manage in the face of social physical and emotional challenges of life’. It exists of six dimensions including (1) bodily functions, (2) mental functions and perception, (3) spirituality or existentialism, (4) quality of life, (5) social and societal participation, and (6) daily functioning [41]. The concept of positive health was introduced to participants during the interview as a way to look at health.

The interview guide addressed the following general topics: health, lifestyle, and lifestyle counselling. The questions were made suitable for patients with limited health literacy by writing them in B1 language level and they were checked by the language ambassador. To further support understanding, we made use of visual aids (Additional file 2) during interviews to complement oral information. After conducting a first pilot interview, the interview guide was adapted to improve flow and understandability. This pilot interview was also included in the analysis. All interviews were audio recorded and transcribed verbatim by BvA and NF, assisted by the tool Amberscript.

### Data analysis

Data analysis was performed using reflexive thematic analysis as described by Braun & Clarke [42, 43]. This approach allows for a deep understanding of participants’ experiences and needs and emphasizes the researcher’s active role and subjectivity in the analysis. The codes and themes generated reflect the researchers’ interpretations. We followed the six steps of reflexive thematic analysis within an inductive, interpretative approach including: (1) familiarizing with the data, (2) generating initial codes, (3) grouping codes in potential overarching themes, (4) reviewing themes, (5) specifying and describing themes and (6) producing the report [42, 43]. Familiarizing with the data was thoroughly conducted by BvA by reading all transcripts. AM read half of the transcripts and other team members read a few transcripts. BvA generated the initial codes with use of Atlas.ti 23. We organized a first brainstorm meeting for the construction of themes with five members of the research team. We then organized a theme reviewing meeting with AM, BvA and two other team members, with further input provided through written feedback from other team members. Steps five and six were led by BvA and AM, in close consultation with the entire research team, both in writing and through meetings. This collaborative and interpretative process aligns with the principles of reflexive thematic analysis, which values depth, subjectivity, and transparency over interrater reliability [43]. Upon completion of the analysis, all themes and quotes were translated into English by a native speaker.

### Research team description

The research team was composed of highly educated female researchers with a Dutch background, ranging in age from 25 to 55 years old. The team included a PhD student (AM), a junior researcher (BvA), a research assistant (NF), assistant professors (LtB, BG, MH), a general practitioner and associate professor (KvA), professors of applied sciences (WK, BG), and a full professor (IS). Together, they had expertise in the field of prevention, health promotion, health psychology, oncology, primary care, supportive care, participant engagement, nursing, nutrition, and qualitative research. Most of them had experience with cancer through their relatives and profession. The research consortium of the GLINK project, comprising healthcare institutions, dietitians, and lifestyle coaches, contributed to the study design and recruitment. There were no prior relationships between the interviewing researcher and the participants.

### Ethical and quality considerations

All participants received an information letter about the study and were given the opportunity to ask questions before or at the start of the interview. All participants provided written informed consent to participate in the study. Transcripts were pseudonymized by removing identifying characteristics from the data. Audio recordings were deleted after transcription. This study was approved by the Ethical Commission Research (In Dutch: ECO) of the Christian University of Applied Sciences in Ede, review number 29.06/24. The Journal Article Reporting Standards for Qualitative Research (JARS-Qual) [44] were considered in reporting the study. ChatGPT was used as a tool to assist in improving the English and sentence structure of the article.

## Results

In total, 18 cancer survivors with different types of cancer were interviewed, 5 males and 13 females. All participants were above the age of 40 years, with an average age of 58.7 years old (SD 8.2). Level of education, health literacy, and place of residence in the Netherlands varied among participants. Detailed participant characteristics are presented in Table 1.

**Table 1:** Participant characteristics (*n* = 18)

Participant	Gender	Age category	Region of the Netherlands	Level of education*	Estimated level of health literacy**	Primary cancer	Treatment type received	Treatment intent
P1	Female	60–69	Central	UAS	Average-high	Fallopian tube	Surgery	Curative
P2	Female	40–49	West	UAS	Average-high	Breast	Surgery, chemotherapy, hormone therapy	Curative
P3	Female	50–59	East	VET	Average-high	Thyroid, with metastasis to breast and lung	Surgery, radiotherapy, immunotherapy, hormone therapy	Unclassifiable
P4	Female	50–59	South	UAS	Average-high	Breast	Surgery, chemotherapy, hormone therapy	Curative
P5	Male	60–69	East	UAS	Average-high	Kahler	Surgery, immunotherapy	Palliative
P6	Female	50–59	West	UAS	Average-high	Breast	Surgery, radiotherapy	Curative
P7	Female	50–59	West	RU	Average-high	Lymphoma	Radiotherapy	Palliative
P8	Female	50–59	West	VET	Limited	Breast	Surgery, radiotherapy	Curative
P9	Female	60–69	East	UAS	Average-high	Uterus	Surgery	Curative
P10	Female	50–59	West	RU	Average-high	Breast	Surgery, radiotherapy, chemotherapy, immunotherapy	Palliative
P11	Male	50–59	East	UAS	Average-high	Testicle	Surgery, chemotherapy	Curative
P12	Female	40–49	East	UAS	Average-high	Breast	Surgery, radiotherapy, chemotherapy	Curative
P13	Male	70–79	East	UAS	Limited	Prostate + melanoma	Surgery, radiotherapy, immunotherapy	Curative
P14	Male	60–69	South	VET	Limited	Esophagus	Surgery, radiotherapy, chemotherapy	Curative
P15	Female	50–59	East	VET	Limited	Stomach	Surgery, chemotherapy, immunotherapy	Curative
P16	Male	70–79	South	RU	Average-high	Testicle	Hormone therapy	Palliative
P17	Female	60–69	Central	VET	Limited	Kidney	Surgery	Curative
P18	Female	50–59	East	VET	Limited	Breast	Surgery, radiotherapy, chemotherapy	Curative

*Level of education: *VET *Vocational Education and Training, *UAS *University of Applied Sciences, *RU *Research University

**Level of health literacy was estimated by the researcher (BvA) using an adapted checklist from Pharos (Additional file 1)

**Fig. 1:**
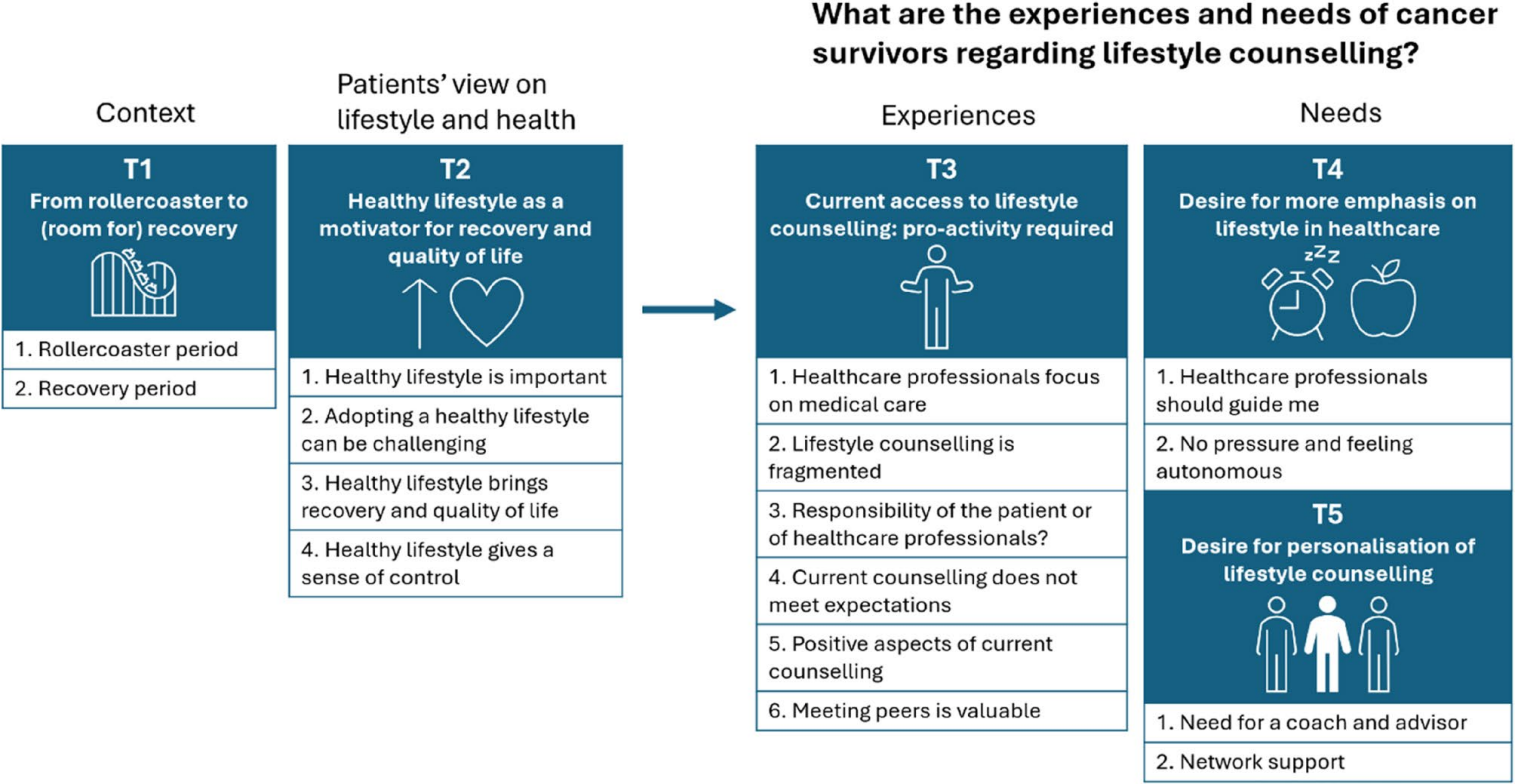
Overview of the five generated themes and their subthemes

Five main themes were generated from the data, including: (1) From rollercoaster to (room for) recovery; (2) Healthy lifestyle as a motivator for recovery and quality of life; (3) Current access to lifestyle counselling: pro-activity required; (4) Desire for more emphasis on lifestyle in healthcare; and (5) Desire for personalisation of lifestyle counselling (Fig. 1). Themes 3, 4, and 5 are directly related to the research question on lifestyle counselling, as they reflect the experiences and needs of cancer survivors. Themes 1 and 2 describe participants’ experiences and needs that are indirectly connected to lifestyle counselling but offer valuable insights for its design and implementation. An overview of the generated themes and subthemes is shown in Fig. 1.

### Theme 1. From rollercoaster to (room for) recovery

Although manifested in many different forms, cancer was commonly experienced by participants as a highly unpredictable and impactful disease, leading to feelings of vulnerability, anxiety, and insecurity. Participants indicated that after diagnosis, two important periods of coping with cancer diagnosis occur, which often occur sequentially but may also overlap: (1) the rollercoaster period, and (2) the recovery period.

#### Rollercoaster period

According to participants, the rollercoaster period often occurred during diagnostics and shortly after diagnosis and was commonly characterized by the focus on the medical aspects of their disease. Participants were busy with undergoing treatments and strived for recovery or control over the disease. Although treatment in hospitals was frequently experienced as a streamlined process by participants, many participants mentioned that it felt like a rollercoaster, causing feelings of insecurity, stress, restlessness, powerlessness, and limited autonomy and freedom.

*“After the diagnosis*,* you’re mostly just going through the motions. I really didn’t like that… Your schedule and life are basically dictated by the hospital. You lose nearly all your control and direction over what happens—it’s just taken from you*,* and you have no say in it”* [P4, woman, 54 years, with average-high health literacy].

#### Recovery period

Participants indicated that the recovery period often occurred later; either during or after treatment. They characterized this period by the process of moving beyond the medical context, finding a new balance in life, and rediscovering and trusting oneself, both physically and mentally. During this recovery period, participants often regained autonomy compared to the rollercoaster period. Participants indicated that they were able to re-integrate in society, including their social lives and work.

*“I’ve also noticed that when you’re in the middle of diagnostics and treatment*,* it feels like you’re racing through everything… But the moment you hear that everything is okay is when the real processing starts”* [P1, woman, 60 years, with average-high health literacy].

At the same time, participants experienced finding a new balance after the rollercoaster period challenging for a number of reasons. First, participants were confronted with a lack of daily structure after a very busy period, making them feel lost. In addition, they experienced physical and mental consequences of cancer and its treatment, such as extreme and unpredictable fatigue. They also had to discover their, often altered, physical and mental capacity, possibilities, and limits again, since they mentioned that they were not the same person as they were before they had cancer. This continuously required well-thought decisions in every aspect of life, needing participants to set needed boundaries and prioritize rest, relaxation, and sleep.

*“Cancer-related fatigue is uncontrollable. It can suddenly hit you out of nowhere and completely overwhelm you”* [P7, woman, 54 years, with average-high health literacy].

### Theme 2. Healthy lifestyle as a motivator for recovery and quality of life

#### Healthy lifestyle is important

All participants mentioned the importance of maintaining a healthy lifestyle for their overall health, both before and after their cancer diagnosis. Many participants reported that they were already interested in and had a relatively healthy lifestyle before their diagnosis but became more consciously focused on maintaining or improving their lifestyle after their diagnosis. Lifestyle components were viewed by participants as interconnected, forming a coherent whole influencing the dimensions of positive health, which motivated them to make lifestyle changes, even when they were challenging. This awareness was not limited to those with high health literacy, as participants generally recognized the connection between lifestyle, health, quality of life, and overall wellbeing. However, personal definitions of healthy lifestyle varied. For example, some emphasized the importance of autonomy and freedom of choice and closely associated a healthy lifestyle with enjoying life. Finding a balance between healthy habits and personal enjoyment was considered essential for quality of life.

*“A healthy lifestyle is important*,* but I also think you should enjoy life now and then. You’re still alive*,* so you shouldn’t put everything aside”* [P17, woman, 65 years, with limited health literacy].

#### Adopting a healthy lifestyle can be challenging

While many participants recognized the importance of a healthy lifestyle, some participants did not experience urgency to adopt healthier habits. Various barriers made it challenging to adopt or maintain a healthy lifestyle, including physical limitations, such as pain or fatigue. Additionally, stress, time constraints, procrastination, and temptations further hindered participants’ effort to adopt a healthy lifestyle.

*“The more I walk*,* the more it hurts”* [P18, woman, 55 years, with limited health literacy].

*“Cooking and baking and such*,* exercising*,* sleeping. That alone is almost a full-time job.”* [P3, woman, 59 years, with average-high health literacy].

#### Healthy lifestyle brings recovery and quality of life

Participants mentioned that personal characteristics, such as mindset, self-control, and intrinsic motivation shaped their adoption and maintenance of a healthy lifestyle. Participants had varying motivations for healthy lifestyle engagement. A commonly noted motivation was its role in their recovery and promoting their quality of life. Cancer and its treatment highly impacted participants’ physical functioning, and thereby also negatively influenced the other aspects of positive health: participation in society, meaning of life, quality of life, mental wellbeing, and daily functioning. Participants’ perspectives on health often shifted towards a deeper appreciation of physical health as a fundamental aspect of health and life.

*“I have a lot of self-motivation*,* but most people just need a helping hand”* [P16, man, 71 years, with average-high health literacy].

*“If it doesn’t work out one day*,* well*,* we’ll just start again tomorrow. That’s it. New mindset.”* [P8, woman, 59 years, with limited health literacy].

*“I feel extremely limited by my body*,* illness*,* treatments*,* and their effects*,* which have taken away part of my quality of life—especially my freedom to do what I want”* [P3, woman, 59 years, with average-high health literacy].

A healthy lifestyle was viewed as a way to improve physical condition, minimize physical complaints, stimulate mental wellbeing, maintain energy and vitality, and so enhance quality of life. Noticing positive effects and seeing progress inspired participants to continue their efforts. However, participants mentioned that full physical recovery did not always occur, and that they felt limited in changing this. This could lead to feelings of powerlessness and loss of freedom, which in turn negatively influenced their quality of life.

*“I think that if you just stay active*,* you’ll also gain more energy”* [P17, woman, 65 years, with limited health literacy].

*“I want to keep my body and mind healthy*,* …*,* with as few physical complaints as possible… That way*,* I can keep cycling*,* walking*,* and*,* if possible*,* even hiking in the mountains—because that is important for my quality of life”* [P9, woman, 66 years, with average-high health literacy].

#### Healthy lifestyle gives a sense of control

Adopting a healthy lifestyle provided a sense of control and stability for some participants in the face of a cancer diagnosis, which was often accompanied by feelings of uncertainty and loss of control. Participants mentioned that engaging in a healthy lifestyle allowed them to focus on aspects of their well-being that were within their control. They could set achievable goals, providing direction and purpose.

*“It feels good to do something that helps your body process all those medications… —it gives you a sense of control*,* knowing you can do something to help”* [P10, woman, 53 years, with average-high health literacy].

### Theme 3. Current access to lifestyle counselling: pro-activity required

#### Healthcare professionals focus on medical care

According to participants, hospital care primarily focuses on medical aspects related to cancer. They indicated that lifestyle considerations were not routinely addressed by healthcare professionals during patient consultations in the hospital. Additionally, participants mentioned that referral to lifestyle counselling by professionals such as a dietitian, physiotherapist, or integrated lifestyle programs, was rarely initiated by healthcare professionals in the hospital. Thus, they highlighted that access to lifestyle counselling currently largely depends on the intrinsic motivation and pro-activity of individual healthcare professionals and patients. Patients commonly experienced the absence of referral to lifestyle counselling as a shortcoming of healthcare.

*“Hospitals mainly focus on the medical side. If you ask for support*,* they’re willing to think along with you. But if you don’t ask*,* it’s unclear if you’ll be offered help. It varies greatly depending on the doctor or nurse specialist”* [P10, woman, 53 years, with average-high health literacy].

*“Despite being strong and disciplined*,* I found coaching myself too hard. I felt like: Hold me for a moment and tighten the ropes I can walk along*,* so I have something to hold onto”* [P3, woman, 59 years, with average-high health literacy].

#### Lifestyle counselling is fragmented

Participants mentioned that the availability of lifestyle counselling differed per type of cancer, region, and hospital in the Netherlands. They mentioned that existing counselling is fragmented and often had to be found by chance, for example through participants’ own personal networks. The search for lifestyle counselling also required insight by participants into their own needs. They experienced this as challenging, especially while simultaneously managing mental and physical challenges.

*“A lot of services are offered around here*,* but they are just scattered fragments you have to stumble upon by chance”* [P2, woman, 45 years, with average-high health literacy].

Participants searched for information related to lifestyle and cancer and sometimes struggled where to find reliable answers to their questions. Some expressed non-evidence based beliefs, especially about nutrition and cancer, such as ‘sugar feeds cancer’, reflecting their search for control and fear to hinder their recovery.

*“There are so many conflicting messages*,* like: Should you boost your immune system or not? Because you might also be boosting the cancer cells”* [P7, woman, 54 years, with average-high health literacy].

#### Responsibility of the patient or of healthcare professionals?

Some participants viewed the search for lifestyle counselling as their own responsibility or as necessity, thus sought for it themselves when they needed and desired it. Other participants were less pro-active and did not search for lifestyle counselling themselves, because they were hesitant or did not need or desire counselling. This group tended to rely more on advice and referrals provided by healthcare professionals in the hospital.

*“I can do my own research too… When you get a diagnosis*,* you can read up on what’s good for you and what suits you”* [P14, man, 61 years, with limited health literacy].

*“It’s not just about handing out a leaflet. It’s about guiding people from the intake through the whole process—checking in on how they’re doing. Hormone treatments make you feel awful*,* and they damage your bones. But lifestyle changes can help enormously—yet*,* almost nothing is done with that”* [P16, man, 71 years, with average-high health literacy].

#### Current counselling does not meet expectations

When provided, lifestyle counselling did not consistently align with participants’ needs and expectations. Some participants held high expectations regarding the advice they would receive but reported that the information provided was too general for them and therefore neither novel nor insightful, as they sought knowledge specific to their type of cancer.

*“The lifestyle advice felt too generic. When I asked if they had nutrition tips specific to my cancer*,* they gave me general advice from the food pyramid. I found that quite shocking”* [P3, woman, 59 years, with average-high health literacy].

Participants also expressed dissatisfaction with professionals providing lifestyle counselling, such as dietitians, physiotherapists, and lifestyle coaches. They often perceived professionals to have limited expertise in oncology and insufficient capacity to empathize with and align to the needs of cancer patients. According to participants, this may potentially result in a lack of flexibility in tailoring health advice to their specific situations and desires.

*“I also went to a physiotherapist who didn’t know anything about cancer or lymphoma. He treated me anyway*,* but in the end*,* it was pointless”* [P7, woman, 54 years, with average-high health literacy].

#### Positive aspects of current counselling

Lifestyle counselling was perceived as particularly beneficial by participants when professionals took a personalised approach and demonstrated a willingness to tailor advice to individual needs and circumstances. Furthermore, lifestyle advice was appreciated by participants when experienced as insightful or novel and when implemented together with professionals by setting realistic goals with clear boundaries. Some participants indicated they received motivational support from their healthcare professional, which they really appreciated and helped them to implement and sustain their lifestyle changes.

*“I wouldn’t keep a diary for a week on my own. So*,* it helps when someone says: ‘Let’s tackle this together*,* map it out*,* and then sit down to see how we can improve things”* [P11, man, 55 years, with average-high health literacy].

*“Well*,* in my case*,* the trainers from [name running program] are just great. They’re really focused on cancer. And you can even message them if you have a question*,* an issue*,* or something related to nutrition.”* [P8, woman, 59 years, with limited health literacy].

#### Meeting peers is valuable

Participants also emphasized the value of peer support. Meeting fellow patients in similar situations easily created a sense of mutual understanding. Participants appreciated the opportunity to exchange experiences, often finding motivation and encouragement in these interactions.

*“One word is enough to understand each other”* [P10, woman, 53 years, with average-high health literacy].

*“Doing it together is fun*,* and knowing that others are counting on you gives you an extra push… For me*,* those exercise sessions with the other women felt like a break from being sick—just enjoying exercise and good company”* [P4, woman, 54 years, with average-high health literacy].

### Theme 4. Desire for more emphasis on lifestyle in healthcare

Most participants expressed a need for increased attention to lifestyle factors related to cancer in healthcare, both during and after treatment phase. They indicated that the relevance of lifestyle should be communicated more clearly with patients. Specifically, participants highlighted the need for (1) guidance of healthcare professionals in identifying their lifestyle-related needs, and (2) the importance of keeping their autonomy and not feeling pressured to engage in lifestyle counselling.

#### Healthcare professionals should guide me

Patients expressed a need for support in identifying their personal lifestyle-related needs, and identifying the resources and support required to address those needs. This was considered valuable by patients irrespective of their level of health literacy. Patients desired more insight into possibilities for lifestyle counselling, by discussing these with healthcare professionals in the hospital setting. They indicated that a more pro-active role of healthcare professionals is needed to improve accessibility and visibility of possibilities. Some participants suggested the idea of a collaborative healthcare team that identifies patients’ needs and provides appropriate help by referring to the right professionals. According to participants, lifestyle counselling should be routinely brought to attention by healthcare professionals in person as part of standard healthcare practice. However, the actual lifestyle counselling interventions were preferred outside the hospital, preferably in local facilities such as local cancer support centres.

*“It would be good if lifestyle support were offered as standard—to see if people would like guidance*,* advice*,* or recommendations”* [P11, man, 55 years, with average-high health literacy].

*“I experience a lot of highs and lows*,* and I think that if you take someone by the hand and consider: yes*,* what do they have*,* what have they had? What other complaints or issues do they have*,* and create a program based on that… I think you can get a lot of support from that.”* [P17, woman, 65 years, with limited health literacy],

#### No pressure and feeling autonomous

While participants appreciated awareness raising and encouragement from healthcare professionals in the hospital setting, they did not want to feel pushed into engaging in lifestyle counselling. They placed great value on their autonomy and freedom in making decisions regarding lifestyle counselling, including its appropriate timing. Overall, participants had more time and mental space to focus on their lifestyle during the recovery period compared to the rollercoaster period and to pre-diagnosis when they were busier with work or other obligations.

Some patients did not express a strong need or desire for lifestyle counselling. They mentioned several reasons such as dissatisfaction with previous lifestyle counselling, beliefs that such counselling would not be beneficial, the belief that maintaining a healthy lifestyle (physically and mentally) is a personal responsibility, and feeling confident and capable to manage their lifestyle without help. Others were sometimes hesitant to allow professionals to determine their actions. They highly valued their autonomy and freedom in making health-related decisions.

*“When I have a sandwich with chocolate sprinkles in the morning*,* the dietitian says it would be better to have one with cheese… I’m going to have it anyway”* [P14, man, 61 years, with limited health literacy].

*“I just want professionals to leave me alone and stop pushing me. I want to do things my way”* [P18, woman, 55 years, with limited health literacy].

### Theme 5. Desire for personalisation of lifestyle counselling

Participants expressed a desire to feel acknowledged and understood. They emphasized that personalising lifestyle counselling is key to fostering this sense of recognition, which in turn increases their motivation to initiate and sustain lifestyle changes. Specifically, participants preferred a person-centred approach over a one-size-fits-all strategy, in which lifestyle counselling is tailored to the individual needs and possibilities of each patient. They desired flexibility and autonomy in selecting the lifestyle themes they wish to focus on, recognizing that these needs may evolve over time. Offering a range of options was suggested as a way to enable participants to choose lifestyle themes that resonate most with them.

#### Need for a coach and advisor

Professionals that provide lifestyle counselling – like dietitians, oncology physiotherapists and lifestyle coaches – play a pivotal role in guiding patients through their recovery and lifestyle adjustments in a personalised way. Many participants emphasized the importance of both the advisory and coaching roles of professionals, in which they not only provide expert knowledge and advice, but also offer practical support tailored to patients’ needs.

Concerning the advisory role, participants expressed a clear need for professionals providing lifestyle counselling (e.g., dietitians and lifestyle coaches) to have expertise in oncology, enabling them to provide tailored lifestyle advice specific to cancer patients and their particular type of cancer, rather than general recommendations. Participants also emphasized the importance of flexibility, expecting professionals to adapt their advice to individual preferences, circumstances and capabilities of patients.

*“Someone who helps you with exercise*,* motivates you*,* and has a bit of knowledge about how to gradually build that up*,* also during phases when you’re still dealing with the fatigue from the radiotherapy and so on.” [P6*,* women*,* 57 years*,* with average-high health literacy].*

Concerning the coaching role, participants valued professionals who co-developed personalised, realistic lifestyle plans for recovery with them. Participants expressed a desire for professionals to motivate them and help to navigate the balance between pushing their limits and pacing themselves appropriately. Participants mentioned that they needed help recognizing their limits and interpret their body’s signals, particularly applicable in lifestyle areas like physical activity, daily patterns, and energy balance.

*“People often say*,* ‘Listen to your body*,*’ but everything feels so different that you no longer speak your body’s language. I’m in the rebuilding phase now*,* and it’s nerve-wracking—how do I even begin?”* [P2, woman, 45 years, with average-high health literacy].

#### Network support

Additionally, patients highlighted the importance of support from their own social environment to navigate life and to initiate and sustain lifestyle changes. Therefore, a greater focus on involving their own social environment in lifestyle counselling was deemed important. Participants indicated to feel supported when family and friends assisted in the management of daily patterns, energy balance and setting boundaries. Also, they felt supported and motivated when they could live a healthy lifestyle together with them. However, patients also noted challenges associated with their social environment. Some experienced a lack of understanding or empathy from those around them. Also, mistaken expectations about quick recovery after treatment could be confronting for patients.

*“The most important thing of all is not home care from an external person*,* but from your own support network.”* [P13, man, 78 years, with limited health literacy].

*“When treatment ends*,* I literally thought: ‘Well*,* that was the last chemo*,* hat off*,* back to work.’ But then I looked in the mirror and realized: ‘This is going to take a while.’ I also noticed people around me reacting the same way: ‘You’re done with treatment now*,* so you’re back*,* right?’ Well*,* no. Absolutely not. It lingers for a long time afterward”* [P2, woman, 45 years, with average-high health literacy].

## Discussion

### Main findings

This study provides insight into the experiences and needs of Dutch cancer survivors regarding lifestyle counselling. The key findings were that patients currently experienced that lifestyle counselling is not structurally embedded in healthcare and often provided in a fragmented manner. They experienced that they must be proactive to access lifestyle counselling, as healthcare professionals in the hospital primarily focus on medical care. Most patients were intrinsically motivated to adopt and maintain a healthy lifestyle as they personally defined it, as it provided a sense of control and enhanced their recovery and quality of life. They also expressed a desire for improved visibility and accessibility of lifestyle counselling. They preferred personalised counselling, tailored to their individual needs and specific type of cancer. According to them, professionals should adopt both an advisory and coaching role, offering lifestyle counselling that is flexible and tailored, which requires expertise in oncology. Additionally, they emphasized the importance of involving their social environment and peers in counselling, as they can provide valuable support and accountability.

### Findings in perspective

Our findings highlight patients’ desire for greater emphasis on lifestyle counselling in healthcare. This aligns with the recent survey conducted by the Dutch Federation of Cancer Patients Organizations [26] which reported that 70% of Dutch cancer survivors wished to discuss lifestyle with a healthcare professional, but 37% reported that cancer related lifestyle changes were actually discussed in the hospital. Lifestyle related help from patients’ general practitioner or practice nurse ranged from 11% for nutrition and physical activity to 44% for smoking [26]. Similarly, a study among endometrial cancer survivors in the UK, stressed the need for lifestyle advice from healthcare professionals in the hospital and referrals to appropriate services [45] and reviews on nutrition and physical activity counselling preferences among cancer survivors also the desire for greater emphasis on lifestyle counselling in healthcare [46, 47].

Our participants reported that the inadequate attention for lifestyle in healthcare was largely due to the medical focus of healthcare professionals in the hospital, suggesting a tension between the formal roles of medical professionals and the expectations patients have of them. Our finding aligns with existing literature on healthcare professionals’ perspectives, showing that they encounter multiple barriers to addressing lifestyle and providing related advice in (oncology) healthcare setting [27, 48–51]. These barriers include limited knowledge of lifestyle and lifestyle guidelines, as well as limited knowledge and skills on how to discuss lifestyle with patients, resulting in discomfort in discussing these topics with patients [27, 49–52]. Additional challenges mentioned include concerns about patient frailty, fear of blame or obstructing autonomy, and potential harm to patient relationships, assumptions about patients’ disinterest, scepticism about the effectiveness of lifestyle advice, time and resource constraints, lack of a good organisational structing for referral, and doubts about their role in providing such advice [48–50, 52]. These beliefs contrast with the needs of patients found in our study. Our results, therefore, advocate for greater integration of lifestyle awareness into routine medical encounters, and specifically the normalization of lifestyle conversations by healthcare professionals in the hospital.

Several initiatives in the Netherlands already aim to integrate lifestyle into healthcare, such as the Leefstijlloket [53] implemented in hospitals and programs like Beter Gezond [54]. However, our findings suggest that many cancer survivors are either unaware of these services, face barriers in accessing them or find that the services do not meet their needs. Normalising low-threshold lifestyle support (initiating conversations about lifestyle, recognising lifestyle-related needs, and referring to appropriate specialists) as an integral component of care, rather than an additional task, may help shift healthcare professionals’ perceptions of their roles and improve accessibility of lifestyle initiatives. To mitigate the risk of limited consultation time as potential barrier to effective lifestyle counselling in care, integration of brief tools to quickly identify patients’ needs, such as the use of the Positive Health spider web [55] and referral pathways to specialised lifestyle professionals could solve this.

Our findings also highlighted the importance of personalised lifestyle counselling that meets patients’ desire to feel acknowledged and understood, a demand also found in other studies among gynaecological [56], colorectal cancer [57] and head and neck cancer survivors [58], and in studies about experiences with lifestyle interventions [59, 60]. Williams et al. [56] emphasized for example, that patients wanted to feel supported by healthcare professionals providing lifestyle counselling (e.g., dietitians, physiotherapists, and lifestyle coaches) by listening well and asking questions, and also considering broader factors influencing their well-being beyond the disease itself.

This need for personalised lifestyle counselling in our study, alongside peer support, was expressed in patients’ preference for authentic counselling tailored to their specific type of cancer and unique circumstances. Interestingly, they mainly expressed a desire for advice, while counselling is much broader. Actual evidence-based lifestyle recommendations for cancer survivors, when provided, were often experienced as too general and did not meet patients’ expectations. This was also found by Anderson et al. [57] in which colorectal patients indicated that information provided was too broad and they were advised to adopt a ‘trial and error’ approach. Patients in our study appeared to believe in the existence of valuable information that did not reach them. At the same time, they struggled where to find reliable information on lifestyle and cancer and had non-evidence based beliefs about lifestyle, especially about nutrition and cancer. They might be more susceptible to more attractive and convincing non-evidence based information. This aligns with the findings of a study among endometrial cancer survivors in the UK who reported that patients sought lifestyle information online after their cancer diagnosis but struggled to identify trustworthy sources [45]. The evidence-based World Cancer Research Fund (WCRF) lifestyle guidelines for cancer survivors [3] are not specifically tailored to this group but are instead the same as those for prevention of cancer and comparable to other non-communicable diseases evidence-based guidelines [61, 62], reflecting quite general lifestyle recommendations. This may explain why patients feel that this information does not meet their expectations. To take patients’ desires into account, it is crucial that lifestyle counselling, while generally based on general recommendations, is tailored to the individual’s personal circumstances and needs, and personal attention is given alongside peer support. For example, personalised lifestyle counselling may include individually adapted coaching on lifestyle themes like nutrition, stress management, sleep, and exercise. The focus and intensity can be tailored to individuals’ fitness level, gradual progression, symptoms such as cancer-related fatigue, and phase of survivorship. The focus and aim could differ per person, ranging from recovery to long-term health maintenance and recurrence prevention.

Our findings indicate that despite most patients recognizing the importance of a healthy lifestyle and are motivated to make changes, various barriers hinder translating this into sustained lifestyle changes, such as cancer-related fatigue. Even patients who indicated to have high self-control or strong internal motivation to engage in a healthy lifestyle struggled to implement lasting changes independently, highlighting the complexity of lifestyle change, especially while dealing with cancer-related fatigue. Besides motivation, other factors also play a role in adopting and maintaining a healthy lifestyle among cancer survivors, such as social support, receiving professional guidance, self-efficacy, and psychological complaints [25]. It is important that healthcare professionals identify and acknowledge barriers such as cancer-related fatigue and provide the right support to increase motivation, self-efficacy and social support, using behaviour change techniques such as goal-setting, action planning and motivational interviewing [63].

Interestingly, our study did not reveal notable differences in lifestyle counselling needs between individuals with limited health literacy (*n* = 6) and those with average to high health literacy (*n* = 12). However, we observed that health literacy skills appeared to play a crucial role in lifestyle counselling even among patients with moderate to high literacy levels. For example, in navigating lifestyle counselling and evaluating reliability of health information. These findings underscore the need for lifestyle counselling tailored to a diverse patient population, with support tailored to lower health literacy, and the importance of incorporating evidence-based behaviour change techniques into lifestyle counselling [64].

Based on our results we formulate several recommendations on how to take into account health literacy in lifestyle counselling for cancer survivors. First, lifestyle counselling should be provided in an accessible and integrated way as the orientation and navigation in lifestyle counselling is difficult for people with low navigational health literacy [65]. Second, it is important to help patients identify trustworthy, evidence-based information to counter misconceptions about cancer and lifestyle and reduce susceptibility to non-evidence based alternatives [66]. Enhancing patients’ critical health literacy skills is essential, enabling them to interpret and analyse (complex) health information and take control over their health [67]. These skills include information processing skills, judgment skills, and self-management skills [67]. Third, healthcare professionals should use clear, understandable and non-patronizing language and visual materials in lifestyle counselling so that patients feel acknowledged and understood [68]. Finally, different perspectives on lifestyle and health, being part of health literacy [69], shapes how people interpret health information and advice. Lifestyle counselling by healthcare professionals should recognize and acknowledge those different viewpoints, engage with patients’ beliefs, and support patients’ autonomy. To implement effective and personalised lifestyle counselling in practice, healthcare professionals require targeted training in motivational interviewing, behaviour change strategies, survivorship care, and health literacy-sensitive communication. Structured educational programs combined with practical tools and ongoing support may improve the quality and personalisation of counselling, addressing common issues of generic or superficial guidance.

### Study strengths and limitations

One key strength of this study is the diversity of the study population, which includes cancer survivors with various types of cancer, geographical locations, ages and stages of disease. This enabled the exploration of a broad range of experiences and perspectives, thereby enhancing the richness of the data. However, a limitation of this diversity is that it provides limited insight into specific subgroups. Our findings primarily reflect findings across the broader cancer survivor population rather than within particular subpopulations with the same characteristics. Another strength is variety in health literacy levels in our study population. However, this was a researcher-identified subjective estimation; we strived to recognize individuals with lower health literacy without using extensive questionnaires to minimize participant burden and support inclusion. However, this approach may limit the precision with which health literacy levels can be interpreted. Another strength is the broad scope on health incorporating the concept of Positive health, and on lifestyle including multiple lifestyle aspects such as sleep and energy balance. This differs from most other studies on lifestyle needs that concentrate solely on nutrition and/or physical activity. Lastly, participation was highly accessible for participants as they could choose between an online or in-person interview. We did not perceive any differences in response between the online and in-person interviews.

A potential limitation of this study is that almost all participants were motivated to make lifestyle changes to improve their health. While our study population may have been relatively highly motivated, there was still diversity in patients’ experiences and needs regarding lifestyle engagement and counselling varied. A contributing factor to the greater overall awareness and engagement with lifestyle changes among our study population could be the increasing collective attention to healthy lifestyle in healthcare in the Netherlands [70]. Another potential limitation is that participants may have provided socially desirable responses, favouring a positive view on healthy lifestyle adjustments. Additionally, collecting extra participant characteristics about the disease, treatment, and sociodemographic information may have offered additional valuable context, but were not systematically collected across the sample. Lastly, throughout the interviews, participants sometimes used general terms when speaking about healthcare professionals providing care to them. Consequently, the term ‘healthcare professionals’ in this manuscript reflects participants’ own broad language, unless otherwise indicated.

### Practical implications and suggestions for further research

Our research showed that improvements in the initiation and referral process of lifestyle counselling are strongly desired by cancer survivors. This is an opportunity for hospital-based healthcare professionals to initiate and normalize conversations about lifestyle with cancer survivors and to refer them to appropriate professionals and services. These healthcare settings include the hospital as well as primary care. Additionally, we recommend that lifestyle referral for cancer survivors should be embedded in an interdisciplinary and integrated network to improve collaboration between public healthcare, primary care and secondary care professionals.

Based on our study findings, we have retrieved starting points that a lifestyle intervention for cancer survivors should comply with, which can be found in Additional file 3. Building on these insights, future research could inform the development or evaluation of targeted integrated lifestyle interventions for cancer survivors.

Further research could explore how lifestyle counselling needs and experiences differ across the disease trajectory of cancer survivors. Also, further research could explore the origins of non-evidence based beliefs about lifestyle among cancer survivors and how these beliefs influence related lifestyle choices. Additionally, further studies could investigate how to bridge the gap between patients’ needs in lifestyle counselling and the barriers healthcare professionals experience in providing lifestyle counselling.

## Conclusions

This study shows the need for stronger integration of accessible, personalised, and integrated lifestyle counselling for cancer survivors within healthcare, with a crucial role for healthcare professionals and patients’ social environment. These findings can inform improvements in the initiation of lifestyle conversations and referral processes and serve as a foundation for developing an integrated lifestyle intervention for cancer survivors.

## Supplementary Information

Supplementary Material 1

Supplementary Material 2

Supplementary Material 3

## Data Availability

The datasets generated during and/or analysed during the current study are available from the corresponding author on reasonable request.

## Abbreviations

IM: Intervention Mapping
HBM: Health Belief Model
