# Supplementary material for: Experiences and needs of Dutch cancer survivors regarding lifestyle counselling: a qualitative study
Source: BMC Cancer. 2025 Nov 12;25:1761. doi: 10.1186/s12885-025-15186-6 (PMC12613877; doi:10.1186/s12885-025-15186-6)
Supplement: Supplementary file 1 — Supplementary Material 1 [file 12885_2025_15186_MOESM1_ESM.docx]

**Additional file 1 – Health literacy estimation checklist**

**Checklist for estimation of healthy literacy level:**

1. Many people have difficulty reading hospital brochures. How is that for you?
2. Many people have difficulty assessing health information. How is that for you?
3. Many people have difficulty to maintain healthy habits. How is that for you?
4. Many people have difficulty filling out (digital) hospital questionnaires. How is that for you?
5. Does anyone ever help you with filling out questionnaires or reading letters?
