# Supplementary material for: Experiences and needs of Dutch cancer survivors regarding lifestyle counselling: a qualitative study
Source: BMC Cancer. 2025 Nov 12;25:1761. doi: 10.1186/s12885-025-15186-6 (PMC12613877; doi:10.1186/s12885-025-15186-6)
Supplement: Supplementary file 2 — Supplementary Material 2 [file 12885_2025_15186_MOESM2_ESM.docx]

**Additional file 2 – Interview guide**

# Interview guide

## Introduction

- Introduce and thank for opportunity to talk
- Introduction to the study
- Explanation and signing of informed consent form

## Health (positive health)

Now I would like to talk to you about health. What do you think of when you hear the word health?

*Showing image of positive health*


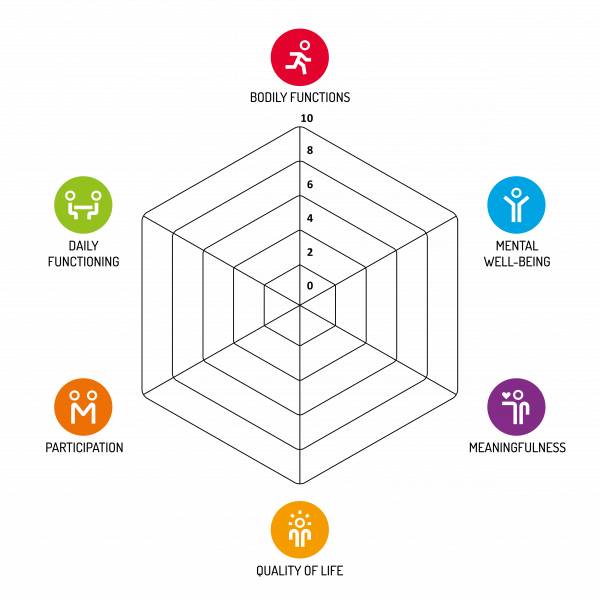


© Institute for Positive Health

Explanation of Positive Health (broad view, much more than just having or not having a disease). Division into 6 components.

When you hear this... and you look broadly at health..

| Topic | Follow-up question |
| --- | --- |
| What is important to you | What makes it important to you |
| Where would you like to see changes | Can you tell me more about this  Who / what do you need to achieve this |

## Healthy lifestyle

Now I would like to talk to you about a healthy lifestyle; what influences health. What do you think of when you hear the term healthy lifestyle?

**
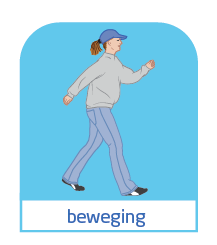
***Using talking cards of various lifestyle items, for example:*

**
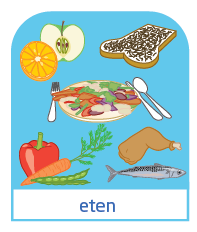

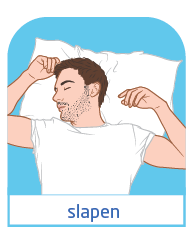
**

© Pharos

| Theme | Main question | Follow-up question |
| --- | --- | --- |
| Importance of a healthy lifestyle | Importance of healthy living (in this situation  *Choosing a talking card*  Which items are important to you? | Impact of cancer (vision)  What makes it important / not important to you?  Has this changed since you became ill? |
| Behaviour | To what extent has your lifestyle changed? (since cancer)  *Choosing a talking card*  Topics you want to change/improve  How to maintain a healthy living | Impact of cancer  Confidence that X will succeed   - Why / why not - Barriers (time, priority, environment, money, fatigue) - Facilitators: who/what is needed (knowledge, environment, skills)   Who / what do you need for this? |
| Doelen | What do you personally want to achieve by living healthier?  Write down goals | Behaviour goal:  Health goal:  Life goal: |

## Lifestyle counselling

Now I would like to talk to you about the counselling you receive regarding your lifestyle during and after your cancer treatment. What do you think of when you hear lifestyle counselling? Think of things like exercises with a physiotherapist, tips on nutrition, or talking to someone about how you feel.

| Onderwerp | Vervolgvraag |
| --- | --- |
| Importance of help/advice/guidance in changing lifestyle (need) | What makes this important/not important to you (impact of cancer) |
| Experiences with lifestyle counselling during/after cancer treatment | What kind of counselling   - By whom - When (during/after treatment) - How often - Offered / self-sought - What went well / not well |
| What kind of guidance do you need to achieve your personal goals (behavior, health) | Where do you mainly need help:   - Which lifestyle themes - Knowledge: learning - Skills: improving - Overcome barriers - How does help look like: who/what is needed - Environment: social + professional |
| Standard guidance offered during/after cancer treatment | How can the current offer be improved Combined program/focus on one lifestyle factor |

## Conclusion

- Anything else they would like to discuss
- Thanking for time and giving voucher
- Reference to aftercare
