# Supplementary material for: Experiences and needs of Dutch cancer survivors regarding lifestyle counselling: a qualitative study
Source: BMC Cancer. 2025 Nov 12;25:1761. doi: 10.1186/s12885-025-15186-6 (PMC12613877; doi:10.1186/s12885-025-15186-6)
Supplement: Supplementary file 3 — Supplementary Material 3 [file 12885_2025_15186_MOESM3_ESM.docx]

**Additional file 3 – Recommendations for the development of a lifestyle intervention for cancer survivors**

**Recommendations for the development of a lifestyle intervention for cancer survivors**

| **TIMING AND PERSONAL NEEDS**   - **Flexible timing:** often not in rollercoaster period but in recovery period: more time and mental space - **Personalized lifestyle counseling is important**: desire to feel acknowledged and understood - **Not everyone desires lifestyle counselling**: some feel confident and capable to manage it independently, others are hesitant to allow professionals to determine their actions - **Arguments for lifestyle change for people who doubt can include:** providing sense of control, or working on recovery and quality of life | **ACCESSIBILITY AND LOCATION**   - **Provision of counselling outside the hospital** - **Accessibility important:** in patients’ region - Not only digital sessions but also **in-person meetings** - Make use of **cancer walk-in centers** to provide (informal) support |
| --- | --- |
| **ROLE OF HEALTHCARE PROFESSIONALS**   - **More attention to lifestyle factors in healthcare by healthcare professionals in the hospital and primary care (nurses and doctors)**   - Counselling in personal needs and referral to counselling that addresses these needs - **Professionals providing lifestyle counselling** (dieticians, physiotherapists, lifestyle coaches) **should:**   - Have expertise in oncology   - Adapt their service to individual preferences, circumstances and capabilities   - Make lifestyle plans together with the patients   - Motivate patients   - Help navigate the balance between pushing their limits and pacing themselves - **A collaborative healthcare team** that works together and refers to each other provide patients with the right support - **Accessibility:** low-threshold contact with professionals - **Provide insight in and offering different possibilities for counselling** | **CONTENT AND APPROACH OF LIFESTYLE INTERVENTIONS**   - **Assessing multiple lifestyle factors in an integrated way**: these are interconnected and impact overall health - **Autonomy and freedom of choice is important** - **Behavior change is difficult** (due to physical limitations, time constraints, procrastination, temptations) 🡪 provide the right support for this by using behavior change techniques - **Make use of facilitators:** their interest in lifestyle topics, support from professionals and own social network - **Enhance critical health literacy skills to recognize reliable information from trustworthy sources** - **Group based activities:** peer support is valuable as it creates a sense of mutual understanding - **Mixed groups:** Different types of cancer in both curative and palliative phases. - **Involve patients’ social environment:** they can assist in the management of daily patterns and energy balance. Also living a healthy lifestyle together with them brings support and motivation |
| **HOW TO TAKE INTO ACCOUNT HEALTH LITERACY**   - **Accessible and integrated lifestyle counselling:** to support patients with the orientation and navigation in lifestyle counselling - **Enhance patients’ critical health literacy skills:** to identify trustworthy, evidence-based information - **Effective patient-provider communication:** use clear, understandable and not patronizing language and the use of visual materials - **Recognize and acknowledge different viewpoints on lifestyle and health** | |
